# Infection Prevention and Device Pocket Management in Cardiac Implantable Electronic Devices: Emerging Strategies

**DOI:** 10.1007/s11886-026-02436-7

**Published:** 2026-10-15

**Authors:** Benito Jonas Baldauf, Kerstin Bode, Ojan Assadian, Marzia Giaccardi, Pasquale Notarstefano, Ahmet Adiyaman, Martin Hemels, Ernest W. Lau, Hendrik Bonnemeier

**Affiliations:** 1 Life Sciences, University of Applied Sciences Bremerhaven, An der Karlstadt 8, Bremerhaven, 27568 Germany; 2 Medical Faculty, Christian-Albrechts-University Kiel, Kiel, Germany; 3 Department of Electrophysiology, Heart Center Leipzig at University of Leipzig, Leipzig, Germany; 4 Institute for Skin Integrity, University of Huddersfield, Queensgate, Huddersfield HD1 3DH UK; 5 University Hospital Wiener Neustadt, Corvinusring 3–5, Wiener Neustadt, 2700 Austria; 6 Department of Cardiology, Santa Maria Annunziata Hospital, Florence, Italy; 7 Cardiology Unit, Meyer University Hospital IRCCS, Florence, Italy; 8 Department of Cardiology, San Donato Hospital, Arezzo, Italy; 9 Department of Cardiology, Isala Hospital, Zwolle, The Netherlands; 10 Department of Cardiology, Rijnstate Hospital, Arnhem, The Netherlands; 11 Department of Internal Medicine and Cardiology, Asklepios Klinikum Schildautal, Seesen, Germany

**Keywords:** Cardiac implantable electronic devices, CIED infection, Infection prevention, Pocket management, Biofilm, Antibacterial envelope, Leadless pacemaker, Subcutaneous ICD, Precision medicine, Risk stratification

## Abstract

**Purpose of Review:**

Cardiac implantable electronic device (CIED) infection remains one of the most serious complications of device therapy, causing significant morbidity, mortality, healthcare utilization, and costs. Despite advances in device technology and preventive strategies, infection rates have not declined and may be increasing. This review summarizes current evidence on CIED infection prevention and device pocket management, with a focus on risk-based prevention, procedural strategies, and emerging technologies.

**Recent Findings:**

Increasing evidence supports individualized infection-prevention strategies based on patient-, procedure-, and device-related risk factors. Contemporary approaches extend beyond peri-procedural antibiotic prophylaxis and include structured risk stratification, optimization of modifiable risk factors, meticulous pocket management, prevention of pocket hematoma formation, taurolidine containing adjuncts and selective use of antibacterial technologies such as absorbable antibiotic envelopes. Novel device platforms including leadless pacemakers, subcutaneous and extravascular implantable cardioverter-defibrillators, and conduction system pacing may further reduce infectious burden in selected patients. Emerging technologies including antimicrobial biomaterials, artificial intelligence-assisted risk prediction, and digital wound surveillance may facilitate precision-based infection prevention.

**Summary:**

CIED infection prevention increasingly requires a comprehensive and individualized approach that addresses the entire contamination - biofilm - infection pathway. Contemporary prevention strategies integrate patient optimization, procedural excellence, pocket management, and selective use of adjunctive technologies. Future advances are likely to shift infection prevention toward precision-based interventions tailored to individual patient risk profiles and device requirements.

**Graphical Abstract:**

**Figure:**
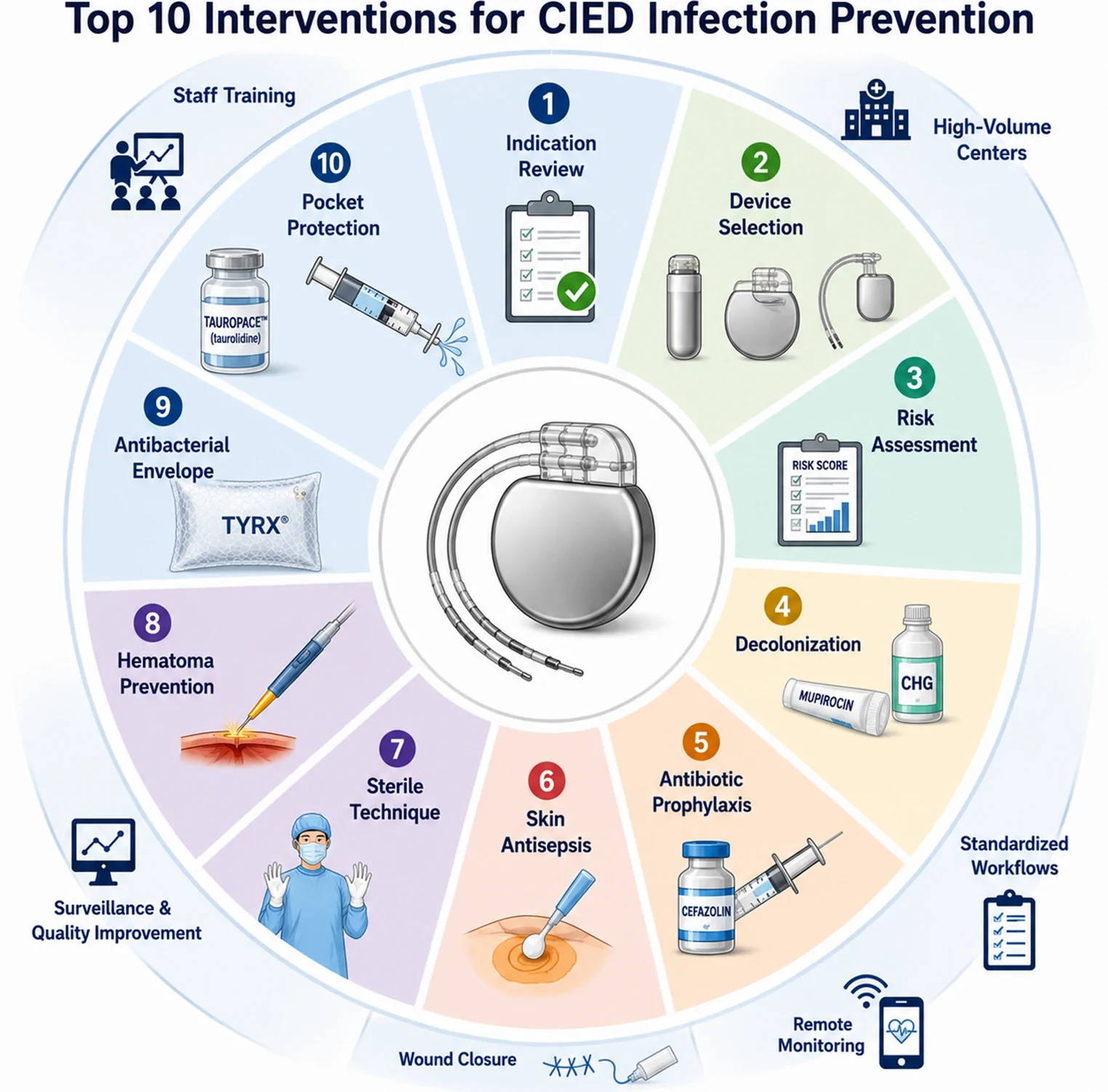
Comprehensive strategy for the prevention of cardiac implantable electronic device infection. Prevention of CIED infection requires a multimodal, patient-centered approach encompassing the entire continuum of care, from patient selection to long-term follow-up. Key components include appropriate indication review, individualized device selection, validated preprocedural risk assessment, targeted decolonization, timely preoperative antibiotic prophylaxis, optimal skin antisepsis, meticulous sterile surgical technique, prevention of pocket hematoma, selective use of adjunctive antimicrobial technologies (e.g., taurolidine-containing solutions and antimicrobial incision drapes), and pocket protection strategies such as antibacterial envelopes in appropriately selected high-risk patients. These procedural measures should be supported by institutional quality initiatives, including standardized workflows, staff education, performance surveillance, treatment in experienced high-volume centers, and remote monitoring when appropriate. Together, these complementary interventions address patient-, procedure-, device-, and healthcare system–related risk factors to minimize the incidence of CIED infection and improve clinical outcomes

## Introduction

The use of CIEDs, including permanent pacemakers, implantable cardioverter-defibrillators (ICDs), cardiac resynchronization therapy (CRT) systems, and emerging leadless and extravascular technologies, has increased substantially over time [1, 2]. This expansion reflects both an aging population with increasing cardiovascular disease burden and continued advances in device-based therapies [3]. Although modern CIED therapy provides substantial improvements in survival, symptom control, and quality of life, infection remains the most frequent complication of implantation and long-term device management [4, 5].

The clinical consequences of CIED infection extend far beyond the implant pocket. Device-related infection necessitates complete system extraction, prolonged antimicrobial therapy, repeated interventions, and extended hospitalization [6]. Mortality remains substantial despite advances in extraction techniques and antimicrobial treatment [7]. Importantly, the economic burden associated with CIED infection continues to rise, driven by increasing procedural complexity and growing numbers of device recipients [8].

Historically, infection-prevention efforts have focused primarily on peri-procedural antibiotic prophylaxis and sterile surgical technique. While these interventions remain fundamental, contemporary understanding of CIED infection has evolved considerably. Increasing evidence suggests that infection prevention should be viewed as a continuous process extending from patient selection and pre-procedural optimization to implantation technique, device selection, pocket management, and post-procedural surveillance [9].

Advances in biofilm biology have transformed our understanding of device-related infection. Once microbial adherence to implanted hardware occurs, mature biofilm formation may render both host immune defenses and antimicrobial therapies substantially less effective [10]. Consequently, preventive interventions are most effective when implemented before bacterial colonization becomes established [11].

The concept of precision infection prevention has therefore emerged. Rather than applying identical preventive measures to all patients, contemporary approaches employ individualized strategies guided by patient-specific risk factors, procedural complexity, and device characteristics. Simultaneously, technologies including taurolidine containing adjuncts [12], antibacterial envelopes [13], leadless pacing systems, extravascular defibrillation platforms [14], antimicrobial biomaterials [15], and artificial intelligence-based risk prediction tools are reshaping the infection-prevention landscape.

This review summarizes contemporary evidence regarding CIED infection prevention and device pocket management, emphasizing the biological mechanisms underlying infection, risk-adapted prevention strategies, and emerging technologies that may further reduce infectious complications in future practice.

## Literature Review Methodology

A targeted literature review was performed to identify contemporary evidence regarding infection prevention and device pocket management in patients undergoing CIED procedures. PubMed/MEDLINE, Embase, and the Cochrane Library were searched for studies published between January 2016 and June 2026 using combinations of terms related to CIED infection, infection prevention, device pocket management, antibacterial technologies, wound closure, hematoma prevention, leadless pacing, and implantable cardioverter-defibrillator therapies.

Priority was given to randomized controlled trials, prospective registries, large observational studies, systematic reviews, meta-analyses, and contemporary guideline and consensus documents from major professional societies, including the European Society of Cardiology, European Heart Rhythm Association, Heart Rhythm Society, American Heart Association, and American College of Cardiology. Landmark studies published before 2016 were included when considered essential to contemporary practice.

Given the limited availability of dedicated CIED-specific evidence for several aspects of device pocket management, relevant data from adjacent surgical disciplines were considered when biologically plausible and clinically applicable. Evidence was synthesized narratively with emphasis on risk stratification, pre-procedural optimization, pocket management, antimicrobial technologies, emerging device platforms, and future directions in precision infection prevention.

## Biofilm Biology and CIED Infection

**Fig. 1:**
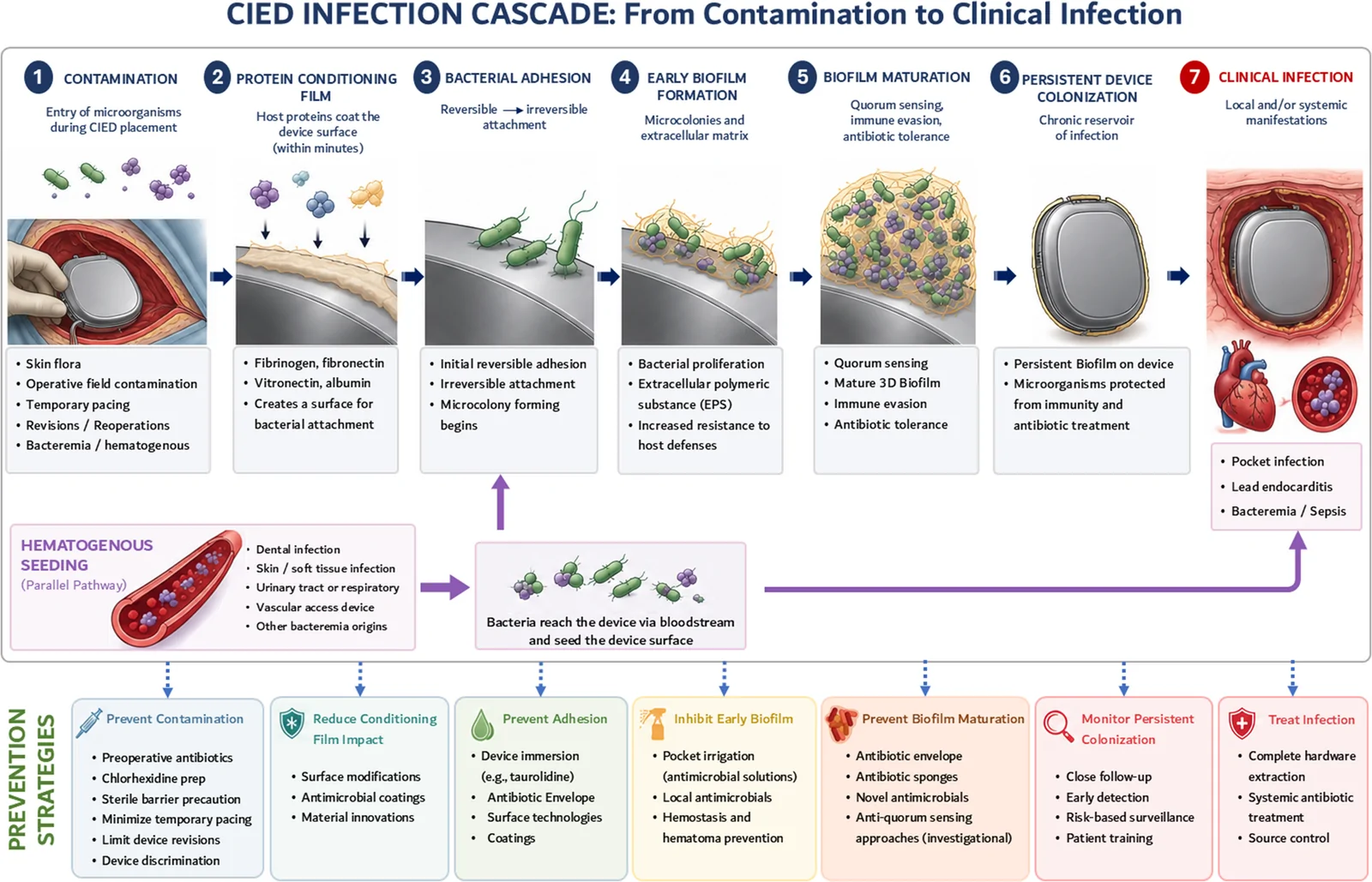
CIED infection cascade from perioperative contamination to established clinical infection and corresponding opportunities for targeted prevention. During device implantation, microorganisms may contaminate the surgical field and rapidly encounter a host-derived protein conditioning film that forms on the device surface within minutes, facilitating bacterial adhesion. Following initial reversible and subsequent irreversible attachment, bacteria proliferate, produce extracellular polymeric substances (EPS), and establish an early biofilm that matures into a three-dimensional structure characterized by quorum sensing (a cell-density-dependent bacterial communication system in which diffusible signaling molecules trigger coordinated changes in gene expression once a critical population threshold is reached), immune evasion, and increased antimicrobial tolerance. Persistent biofilm colonization on the device creates a chronic reservoir of infection that may ultimately manifest as pocket infection, CIED-related infective endocarditis, bacteremia, or sepsis. In parallel, hematogenous bacteremia originating from distant infectious foci can seed the device surface and initiate biofilm formation independently of perioperative contamination. The lower panel highlights stage-specific preventive strategies aimed at interrupting the infection cascade, including meticulous sterile technique, preoperative antibiotic prophylaxis, antimicrobial surface technologies, taurolidine-containing antimicrobial solutions, antibacterial envelopes, local antimicrobial approaches, and risk-based surveillance to prevent progression to clinically manifest CIED infection

The pathogenesis of CIED infection is fundamentally linked to the interaction between implanted foreign material and microorganisms [16]. Unlike many conventional surgical site infections, device-related infections are characterized by the ability of microorganisms to adhere to implanted surfaces and establish highly organized microbial communities known as biofilms [11, 17]. Understanding biofilm biology is therefore essential because most contemporary infection-prevention strategies ultimately target one or more stages of the contamination - adhesion - biofilm - infection cascade. Viewed through this framework, preventive interventions become mechanistically linked rather than isolated procedural measures. Microbial contamination may occur at several stages during the lifetime of a CIED system (Fig. 1) [18].

The most common mechanism involves peri-procedural contamination occurring during initial implantation, generator replacement, up- or downgrade procedures, or lead revision [19]. Even under optimal sterile conditions, complete elimination of exposure to microorganisms is impossible [20]. Skin commensals remain the predominant source of infecting organisms, with coagulase-negative staphylococci and *Staphylococcus aureus* accounting for the majority of infections [21]. A second mechanism involves hematogenous seeding of previously implanted hardware during episodes of bacteremia. Although less common, this pathway may result in lead-related endocarditis [22]. Repeated interventions further amplify risk through disruption of established tissue barriers and potentially chronically colonized pockets [23].

The transition from transient contamination to clinically significant infection involves several sequential biological steps. Immediately following implantation, host proteins rapidly coat device surfaces, creating a conditioning film. Microorganisms expressing surface adhesion molecules can subsequently bind to these protein-coated surfaces and initiate colonization [24]. Following attachment, microorganisms produce extracellular polymeric substances that form the structural matrix of a developing biofilm. This matrix anchors bacterial communities to device surfaces while simultaneously providing protection from environmental threats. As the biofilm matures, bacterial communication through quorum sensing (a cell-density-dependent bacterial communication system in which diffusible signaling molecules trigger coordinated changes in gene expression once a critical population threshold is reached) promotes biofilm maturation and adaptation to environmental stress. This contributes to extracellular matrix production, altered bacterial metabolism, immune evasion, and increased tolerance to antimicrobial exposure. Within this protected environment, bacterial communities may persist for prolonged periods without causing overt clinical infection [25].

Biofilm formation has several important clinical consequences. First, antibiotic penetration into mature biofilms is incomplete, reducing the effectiveness of systemic antibiotic therapy. Second, bacteria within biofilms exhibit markedly reduced metabolic activity, rendering them less susceptible to agents that target actively dividing organisms. Third, biofilms impair host immune responses by physically shielding microorganisms from phagocytic cells and humoral defenses [26]. These mechanisms help explain why established CIED infection will not resolve with antibiotic therapy alone and why complete hardware extraction remains the cornerstone of treatment for device-related infections [4]. Thus, biofilm biology also helps explain why prevention is substantially more effective than treatment: once mature biofilm becomes established, eradication becomes difficult regardless of antimicrobial strategy.

Virtually every preventive intervention employed during CIED implantation targets a specific stage of the contamination - biofilm - infection pathway. Pre-procedural decolonization reduces microbial burden before contamination occurs [27]. Antibiotic prophylaxis limits bacterial survival during the period of greatest vulnerability [28]. Meticulous sterile technique minimizes opportunities for microbial transfer [29, 30]. Hematoma prevention reduces the creation of nutrient-rich environments that facilitate bacterial proliferation [31]. Antibacterial envelopes and device surface decontamination seek to inhibit microbial adherence and early biofilm development [32, 33]. Finally, leadless and extravascular technologies reduce the amount of endovascular implanted foreign material available for hematogenous seeding [34, 35]. Accordingly, contemporary infection prevention should not be viewed as a collection of isolated interventions but rather as a coordinated effort to interrupt multiple stages of the biofilm lifecycle before clinically significant infection becomes established.

## Risk Stratification and Precision Prevention

The next evolution of CIED infection prevention is likely to move beyond simple risk prediction toward individualized preventive pathways. Rather than asking whether a patient is at increased risk, our strategies will need to identify which preventive intervention is most likely to reduce that risk.

In this framework, risk scores become actionable clinical tools that guide decisions regarding antibacterial envelope utilization, device selection, antimicrobial pocket and device management strategies, surveillance intensity, and allocation of preventive resources.

### Pre-Procedural Optimization

Preventive interventions implemented before implantation may be particularly effective because they target the earliest stages of the contamination – biofilm - infection cascade. Reduction of microbial burden, optimization of host defenses, and mitigation of modifiable procedural risk factors collectively decrease the likelihood that bacterial contamination progresses to clinically significant infection. Modifiable patient related risk factors, such as medication, glycemic control and others are discussed in the respective section.

#### Staphylococcus Aureus Screening and Decolonization

Staphylococcus aureus remains one of the most virulent pathogens responsible for CIED infection and device-related endocarditis, while coagulase-negative staphylococci continue to account for the majority of indolent pocket infections [36]. Nasal carriage of methicillin-resistant S. aureus is a well-established risk factor for postoperative infection across multiple surgical specialties and represents a potentially modifiable source of peri-procedural contamination [37]. Evidence from cardiac surgery, orthopedic surgery, and other implant-based procedures has demonstrated that targeted decolonization strategies can reduce surgical site infections. The most commonly employed regimen combines intranasal mupirocin administered for several days before the procedure with chlorhexidine-based body cleansing [38]. Although dedicated CIED-specific evidence remains limited, many centers have adopted decolonization protocols for patients considered at elevated infectious risk [39]. Routine universal screening has not been uniformly implemented across all healthcare systems, and questions remain regarding cost-effectiveness and optimal patient selection. Nevertheless, screening and decolonization should be considered in patients undergoing generator replacement, device upgrades, CRT implantation, or other high-risk procedures, particularly in regions with elevated prevalence of MRSA [38, 40].

#### Preoperative Bathing and Chlorhexidine Cleansing

Preoperative bathing with antiseptic agents represents a simple and low-cost intervention that may reduce cutaneous bacterial burden before implantation. Chlorhexidine-based cleansing is the most frequently studied approach and has demonstrated favorable effects on skin decolonization. Although direct evidence specifically in CIED populations remains limited, chlorhexidine bathing is widely incorporated into perioperative infection-prevention bundles and is supported by extensive experience from cardiac, orthopedic, and general surgical practice for individuals at increased risk for infection [38, 39, 41]. Patients should receive clear instructions regarding the timing and method of antiseptic cleansing to maximize adherence and effectiveness.

#### Hair Removal and Skin Preparation

Appropriate skin preparation represents one of the oldest yet most effective infection-prevention strategies. Importantly, hair removal should not be performed routinely unless hair is expected to interfere with the procedure or wound closure. When hair removal is necessary, electric clippers are preferred because razor shaving may create microscopic skin abrasions that increase infection risk. Hair removal should ideally occur immediately before the procedure rather than several hours beforehand.

Alcohol-based chlorhexidine preparations are generally preferred for skin antisepsis owing to their broad antimicrobial activity, rapid onset of action, and sustained residual effect. Meticulous application technique and sufficient drying time are essential to maximize antiseptic effectiveness.

#### Summary

Pre-procedural optimization should be viewed not as a collection of isolated interventions but as a coordinated process aimed at reducing microbial burden and minimizing procedural risk. The greatest benefit is likely achieved when multiple evidence-based measures are included within standardized institutional pathways.

A practical pre-procedural optimization bundle may include screening for active infection, assessment of glycemic control, review of immunosuppressive therapies, individualized management of antithrombotic agents, targeted MRSA decolonization, chlorhexidine-based skin cleansing, and standardized skin preparation protocols.

### Procedural Infection Prevention

#### Procedural Excellence

Despite advances in antimicrobial therapies, antimicrobial technologies, and device design, procedural conduct remains the most important determinant of CIED infection risk. The majority of device infections originate from peri-procedural contamination events occurring during the procedures. Consequently, meticulous procedural execution remains fundamental to infection prevention.

Importantly, procedural infection prevention extends beyond antibiotic administration and sterile attire. Every intraoperative step, from room preparation and skin antisepsis to pocket creation, hardware handling, and wound closure, may influence the probability that contamination progresses to clinically significant infection.

#### Operator Experience

Studies have demonstrated an inverse relationship between operator experience and procedural complications. The association between procedural experience and infection risk likely reflects several mechanisms. Experienced operators generally perform more efficient procedures, minimize tissue trauma, achieve superior hemostasis, and demonstrate greater familiarity with sterile workflows and troubleshooting strategies [42, 43].

Importantly, procedural excellence should be regarded as a collective team competency rather than an individual operator characteristic. Nursing staff, technicians, anesthesiology teams (potentially present), and device specialists all contribute to maintenance of sterile conditions and procedural efficiency.

#### Operating-room environment and airflow

Because contamination of the device or pocket may occur from the operating environment, CIED implantation should be performed in a dedicated operating room or procedure suite meeting the sterile standards applicable to other surgical implant procedures. This includes appropriate ventilation, positive pressure relative to adjacent areas, effective air filtration, limitation of personnel traffic and unnecessary door opening, and appropriate environmental cleaning. General operating-room standards commonly specify at least 15 air changes per hour, although historical CIED-specific recommendations have suggested higher exchange rates. The incremental benefit of laminar airflow or HEPA filtration beyond standard appropriate operating-room ventilation for CIED infection prevention remains uncertain [44, 45], and these measures should therefore not be presented as CIED-specific evidence-based requirements.

#### High-Volume Centers

Institutional procedural volume has emerged as an important determinant of outcomes following CIED implantation. High-volume centers frequently demonstrate lower complication rates. Several factors may contribute to these observations. Experienced centers often maintain dedicated electrophysiology laboratories, specialized implant teams, standardized infection-prevention pathways, and established quality-improvement programs. Furthermore, higher procedural volumes facilitate continuous refinement of workflows and allow more rapid implementation of emerging best practices [46].

#### Standardized Sterile Workflow

Effective infection prevention requires systematic application of sterile principles throughout the entire procedure. Importantly, contamination rarely results from a single major breach but more commonly arises through accumulation of multiple minor deviations from optimal sterile technique.

A standardized sterile workflow should encompass patient preparation, room setup, shelf setup, personnel movement, equipment handling, maintenance of sterile fields, and device implantation. Particular attention should be paid to minimizing unnecessary traffic within procedural suites, reducing airborne contamination (potentially by employing mobile HEPA laminar airflow devices [44]), limiting door openings, reducing procedural interruptions, and ensuring consistent adherence to sterile barriers.

#### Surgical Safety Checklists

Checklists have become integral components of quality-improvement initiatives across numerous surgical disciplines. Beyond ensuring completion of specific tasks, checklists facilitate communication, clarify responsibilities, and promote shared situational awareness among procedural team members [47].

In the context of CIED implantation, procedural checklists may include confirmation of antibiotic administration, verification of skin preparation, assessment of anticoagulation status, availability of required equipment, and review of patient-related infection risk factors (weeks prior).

Importantly, checklists should not be viewed as administrative exercises but rather as practical tools that promote reproducibility and reduce preventable errors. Standardized procedure packs supporting checklist-based approaches have demonstrated improvements in protocol adherence and may contribute to reductions in procedural variability.

#### Antibiotic Prophylaxis

Pre-procedural antibiotic prophylaxis remains one of the most firmly established infection-prevention interventions in CIED implantation [48].

First-generation cephalosporins remain the preferred prophylactic agents in most patients owing to their activity against staphylococcal species and favorable safety profile. Alternative regimens may be required in patients with severe beta-lactam allergy or in adoption to local resistance patterns [28].

The timing of antibiotic administration is critical. Therapeutic tissue concentrations should be achieved before skin incision, emphasizing the importance of structured procedural workflows and checklist-based verification.

While pre-incisional prophylaxis is strongly supported by evidence, routine prolonged postoperative antibiotic administration has not consistently demonstrated additional benefit and is generally not recommended.

#### Incision Protection Strategies

The surgical incision represents the primary interface between the external environment and the implanted device system. Adhesive antimicrobial incision drapes (i.e., iodine-impregnated adhesive drapes) and wound-edge protection strategies have been investigated across multiple surgical specialties with variable results. Although dedicated CIED-specific evidence remains limited, these approaches probably reduce bacterial transfer from surrounding skin into the operative field [29, 30].

#### Hardware Handling Principles

Hardware handling represents an often-underappreciated component of procedural infection prevention. Once sterile packaging is opened, every interaction between device components and the procedural environment creates opportunities for microbial contamination. Contemporary infection-prevention principles emphasize minimizing direct manipulation of generators and leads, limiting exposure of hardware to ambient air, and avoiding unnecessary contact with nonessential surfaces. Device components should remain within sterile packaging until required for implantation and should be introduced into the operative field only when pocket preparation and hemostasis have been completed.

Particular attention should be directed toward generator-pocket interaction. Excessive handling, repeated insertion and removal, and prolonged exposure of hardware before implantation may theoretically increase contamination risk. Although direct evidence remains limited, these principles align closely with established concepts of contamination control and biofilm prevention [25].

Furthermore, all implanted hardware should be handled with awareness that bacterial adherence may occur rapidly following contamination. Because mature biofilm formation is difficult to eradicate once established, prevention of initial microbial transfer remains the most effective strategy.

### From Risk Identification to Guidance of Prevention

Contemporary prevention strategies employ risk stratification not merely to estimate infection probability but to guide individualized preventive interventions. The concept of precision infection prevention recognizes that preventive resources, including taurolidine containing adjuncts, antibiotic envelopes, enhanced peri-procedural protocols, intensified follow-up, or alternative device technologies, should be preferentially allocated to patients with the greatest anticipated benefit. Accordingly, effective risk assessment requires consideration of patient-, procedure-, and device-related factors as well as integration of validated risk prediction models. The most important will be discussed here:

#### Patient-Related Risk Factors

#### Renal Dysfunction

Chronic kidney disease (CKD) and end-stage renal disease represent among the strongest predictors of CIED infection. The risk appears to increase progressively with declining renal function and is particularly pronounced in dialysis-dependent patients. Contributing mechanisms include immune dysfunction, chronic inflammation, frequent vascular access, and repeated healthcare exposure.

Patients with advanced CKD should be considered for intensified infection-prevention strategies, including meticulous hematoma prevention, consideration of antibacterial envelopes during generator replacement or upgrade procedures, and evaluation of leadless pacing systems or ICD systems without transvenous leads when clinically appropriate.

#### Diabetes Mellitus

Diabetes is associated with impaired neutrophil function, delayed wound healing, microvascular dysfunction, and increased susceptibility to bacterial colonization. Poor glycemic control may further increase peri-procedural infectious risk [49].

Pre-procedural optimization of glycemic control should be considered whenever feasible. Enhanced wound surveillance may be particularly valuable in diabetic patients undergoing complex procedures. Preprocedural skin microbiome testing should be considered in presence of diabetic foot ulcers!

#### Immunosuppression

Patients receiving corticosteroids, biological therapies, chemotherapy, any immunosuppressive regimen, or in any immunocompromised state exhibit impaired host defense mechanisms and delayed tissue healing. Similar concerns apply to transplant recipients and patients with active malignancy [50].

Elective procedures should be performed during periods of maximal clinical stability whenever possible. Additional preventive measures, including antibacterial envelopes and intensified follow-up, may be justified in selected high-risk individuals.

#### Previous CIED Infection

Prior device infection is among the strongest predictors of future infection and may reflect persistent host susceptibility, altered local tissue biology, or residual microbial colonization [17]. Reimplantation should incorporate careful reassessment of device indication, consideration of hardware reduction strategies, and selective use of adjunctive preventive technologies.

#### Skin Disorders

Chronic inflammatory skin disorders, recurrent skin infections, and conditions associated with impaired skin barrier function may increase peri-procedural contamination risk [51]. A strategy employing decolonization seems feasible in these patients. Additionally, optimization of dermatologic health before elective procedures may represent a simple but frequently overlooked preventive measure.

#### Procedure-Related Risk Factors

#### Generator Substitution and Device Upgrades

Repeated interventions consistently demonstrate higher infection rates than de novo implantation [52]. Reopening chronic pockets may disrupt previously quiescent biofilms and expose implanted hardware to renewed contamination [53, 54]. Microbial colonization of device surfaces may persist for years without clinical manifestation [55]. Device manipulation may facilitate transition from colonization to active infection.

Generator replacements and upgrades should be regarded as high-risk procedures. Consideration should be given to taurolidine containing adjuncts, antibacterial envelopes, enhanced sterile workflows, and optimization of modifiable risk factors before intervention.

#### Lead Revision

Lead revisions often involve prolonged procedure duration, extensive tissue manipulation, and increased hardware burden. Procedural planning should prioritize minimization of unnecessary hardware (strictly extract abandoned leads) and avoidance of repeat interventions whenever possible. Employment of taurolidine containing adjuncts, antibacterial envelopes, enhanced sterile workflows, and optimization of modifiable risk factors before intervention is recommended.

#### Temporary Pacing

Temporary percutaneous transvenous pacing is consistently associated with increased subsequent infection risk due to the presence of additional intravascular hardware and frequent utilization in critically ill patients [56, 57].

The exit site of the temporary pacing lead should be handled similar to the exit site of a central venous access device with frequent dressing changes and treatment with alcoholic CHX disinfectants under sterile barrier precautions [58].

The duration of temporary pacing should be minimized whenever feasible, and early definitive device implantation should be considered when clinically appropriate.

#### Pocket Hematoma

Pocket hematoma remains among the most important modifiable procedural risk factors. Hematomas may increase infection risk by providing a favorable environment for bacterial proliferation, impairing wound healing, and necessitating repeat intervention [31].

Prevention of hematoma should be considered a primary infection-prevention objective. Anticoagulant therapy such as VKA should be continued (INR 2–3) [59, 60]. Direct oral anticoagulant may be paused prior to the intervention (i.e. last dose the night before the intervention) and started the day after the intervention (i.e. first dose in the morning the day after the intervention). Procedures should be deferred if dual antiplatelet therapy is administered. Meticulous hemostasis, compression strategies, and potentially emerging hemostatic approaches (TXA results when employed during CIED procedures to prevent hematoma formation are inconclusive [61, 62]) are critical components of infection prevention.

Key patient-, procedure-, and device-related risk factors for CIED infection are summarized in Table 1.

**Table 1:** Patient-, procedure-, and device-related risk factors associated with CIED infection

Risk Factor	Pooled Effect Estimate	Significance	Interpretation
PATIENT RISKS			
Previous CIED infection	OR 7.84 (1.94–31.60)	✓	Strongest predictor of recurrent infection; reflects persistent colonization, altered tissue integrity, and host susceptibility
End-stage renal disease	OR 8.73 (3.42–22.31)	✓	Highest-risk comorbidity; immune dysfunction, repeated vascular access, and frequent bacteremia contribute substantially
Renal insufficiency (eGFR < 60 mL/min)	OR 3.02 (1.38–6.64)	✓	Consistently associated with increased infection risk across studies
Permanent central venous access device	OR 5.74 (0.94–34.93)	Borderline	Likely reflects repeated bloodstream access and hematogenous seeding risk
Fever within 24 h before implantation	OR 4.27 (1.13–16.12)	✓	May indicate occult infection; elective procedures should generally be deferred
Corticosteroid therapy	OR 3.44 (1.62–7.32)	✓	Reflects impaired immune response and delayed wound healing
Chronic obstructive pulmonary disease	OR 2.95 (1.78–4.90)	✓	May represent frailty, systemic inflammation, and repeated healthcare exposure
NYHA class ≥ II	OR 2.47 (1.24–4.91)	✓	Marker of advanced cardiovascular disease and increased procedural complexity
Skin disorders	OR 2.46 (1.04–5.80)	✓	Increased bacterial burden and impaired skin barrier function
Malignancy	OR 2.23 (1.26–3.95)	✓	Associated with immunosuppression and healthcare exposure
Diabetes mellitus	OR 2.08 (1.62–2.67)	✓	Impaired wound healing and host defenses
Chronic heart failure	OR 1.65 (1.14–2.39)	✓	Reflects advanced disease burden and repeated interventions
Oral anticoagulation	OR 1.59 (1.01–2.48)	✓	Primarily mediated through hematoma formation
Heparin bridging	OR 1.87 (1.03–3.41)	✓	Strongly associated with pocket hematoma development
Immunosuppressive agents (non-steroid)	OR 1.85 (0.63–5.46)	NS	Biological plausibility despite inconsistent statistical significance
Male sex	OR 1.12 (0.89–1.41)	NS	No convincing association demonstrated
Age	WMD − 1.27 (− 3.08–0.55)	NS	Age alone does not appear to independently increase infection risk, however newer data suggests increased risk in younger individuals [1, 63]
BMI ≥ 25 kg/m²	OR 1.04 (0.63–1.72)	NS	No consistent relationship identified
Smoking	OR 0.48 (0.11–2.08)	NS	Evidence inconsistent
PROCEDURE-RELATED RISKS			
Hematoma formation	OR 8.46 (4.01–17.86)	✓	Strongest modifiable procedural risk factor; prevention should be a major procedural goal
Lead dislodgement/repositioning	OR 6.37 (2.93–13.82)	✓	Necessitates repeat manipulation and prolonged exposure
Inexperienced operator	OR 2.85 (1.23–6.58)	✓	Reflects procedural efficiency and adherence to infection-prevention protocols
Temporary pacing before implantation	OR 2.31 (1.36–3.92)	✓	Potential route for bacterial colonization and bloodstream infection
Device revision/upgrade/replacement	OR 1.98 (1.46–2.70)	✓	Likely related to biofilm disruption and repeat pocket entry
Generator replacement	OR 1.74 (1.22–2.49)	✓	Consistently associated with increased infection risk
Longer procedure duration	WMD 9.89 min (0.52–19.25)	✓	Increased opportunity for contamination and tissue trauma
Absence of antibiotic prophylaxis	OR equivalent 3.13*	✓	One of the most effective preventive interventions
DEVICE-RELATED RISKS			
Epicardial leads	OR 8.09 (3.46–18.92)	✓	Reflect greater surgical complexity and tissue disruption
Abdominal generator pocket	OR 4.01 (2.48–6.49)	✓	Larger pocket size and wound burden contribute to risk
≥ 2 implanted leads	OR 2.02 (1.11–3.69)	✓	Increased foreign-body burden and procedural complexity
CRT device	OR 1.92 (0.90–4.10)	Borderline	Likely reflects greater hardware burden and procedural complexity
Abandoned leads	OR 1.82 (0.86–3.83)	NS	Biological plausibility despite inconsistent evidence
Dual-chamber system	OR 1.45 (1.02–2.05)	✓	Additional lead burden may contribute to risk
ICD system	OR 1.19 (0.84–1.68)	NS	Device type alone does not appear to independently increase risk

*BMI *Body mass index, *CIED *Cardiac implantable electronic device, *CRT *Cardiac resynchronization therapy, *eGFR *Estimated glomerular filtration rate, *ICD *Implantable cardioverter-defibrillator, *NYHA *New York Heart Association, *OR *Odds ratio, *WMD *Weighted mean difference, *NS *Not statistically significant

Data derived from Polyzos et al. [64].

#### Risk Prediction Models

Several risk prediction tools have been developed to facilitate individualized infection-prevention strategies (Table 2).

**Table 2:** Summarizes commonly used risk-stratification models developed to estimate the likelihood of CIED infection based on patient-, device-, and procedure-related characteristics

Score	Key Variables	Intended Use
PADIT	Age, procedure type, renal dysfunction, immunocompromise, prior procedures	Broad procedural risk assessment
BLISTER	Bleeding risk and infection predictors	Identification of high-risk procedural candidates
RI-AIAC	Patient and procedural variables	Infection prediction
Shariff	Clinical comorbidities and procedural complexity	Risk stratification
Kolek	Device-related and patient-related factors	Procedural risk assessment

These scores are intended to support individualized risk assessment, facilitate identification of patients who may benefit from enhanced preventive strategies, and inform clinical decision-making regarding procedural planning and postoperative surveillance [63, 65–68]

*BLISTER *Bleeding Risk and Infection Score for Implantable Electronic Device Procedures, *CIED *Cardiac implantable electronic device, *PADIT *Prevention of Arrhythmia Device Infection Trial, *RI*-*AIAC *Risk Index for Cardiac Implantable Electronic Device Infection

Although discrimination varies among models and no single score has achieved universal adoption, these tools provide a structured approach to identifying patients who may benefit from intensified preventive interventions.

#### Transfer Risk Prediction Into Clinical Action

A persistent limitation of the CIED infection literature is that validated risk models have been used predominantly to estimate infection probability rather than to stratify preventive intervention [69]. Risk prediction achieves clinical value only when it is coupled to a differentiated management pathway. We propose a three-tier framework that maps established risk categories onto graded prevention strategies, distinguishing interventions supported by RCT evidence from those currently supported only by observational or registry data.

*Low-risk patients OR < 2 and PADIT 4 and lower* (female) aged above 60 years undergoing a first implant by experienced operators and procedural time of below 60 min, with preserved renal function, no immunosuppression, no prior device infection, no malignancy, no heart failure, no diabetes, no skin disorder, and receiving a simple single- or dual-chamber system represent the population in whom baseline infection incidence is already low (around 1% in most contemporary series). In this tier, standard antibiotic prophylaxis, proper sterile technique, and scheduled follow-up remain sufficient. Routine employment of taurolidine containing adjuncts may be considered as well as antimicrobial adhesive incision drapes, as these are probably cost effective even for low-risk interventions and do no harm. Escalating prevention further needs to be weighed against the financial burden that comes with its employment as many interventions/devices offer little detectable benefit against a low event rate and are not supported by current guidance or are not cost efficient if employed universally.

*Intermediate-risk patients OR > 2 and PADIT 5–6* (male and/or female aged < 60 years) suffering from diabetes, moderate chronic kidney disease, malignancy, heart failure, COPD, receiving generator replacement, high power devices or more than two leads, or are administered therapeutic anticoagulation, warrant enhanced pre-procedural optimization and deliberate hematoma-avoidance strategy. Here the evidence base is comparatively strong: BRUISE CONTROL [60] demonstrated that continued warfarin, rather than heparin bridging, reduces clinically significant pocket hematoma, and BRUISE CONTROL-2 [59] extended this logic to direct oral anticoagulants, supporting a minimal-interruption strategy over interruption-and-bridge. Since hematoma is the single strongest modifiable precursor of pocket infection, this tier is where periprocedural anticoagulation management yields its greatest preventive return. Taurolidine containing solution adjuncts and impregnated adhesive incision drapes should be considered standard of care, given their favorable safety profile, biological plausibility, encouraging prospective observational evidence and likely cost-effectiveness despite the current absence of randomized trials. Antibacterial envelope use may be considered on an individualized basis.

*High-risk patients PADIT 7 and higher* displaying acute on chronic kidney disease or dialysis dependency, prior CIED infection, immunosuppression, receiving CRT-D upgrade, multiple prior procedures, procedure duration exceeding 60 min or high PADIT/BLISTER scores, carry the highest absolute infection incidence and thus the greatest achievable absolute risk reduction from intensified prevention [63, 68]. Measures discussed prior for intermediate risk patients are mandatory here. Additionally, this is the population in which the WRAP-IT trial demonstrated a significant reduction in major infection with an absorbable antibacterial envelope, and current guidance supports its use preferentially in this tier [70]. Leadless pacing, where anatomically and clinically feasible, offers a mechanistically distinct advantage by eliminating the transvenous lead and subcutaneous pocket and registry data show lower infection rates with leadless systems, particularly in dialysis-dependent and previously infected patients ICD systems with no transvenous portion of their shock coil should be considered where clinically feasible [71]. Intensified postprocedural surveillance and a low threshold for infectious disease consultation complete this tier.

Taken together, these measures support a comprehensive, risk-informed strategy extending from patient selection and pre-procedural risk assessment through procedural infection prevention and post-procedural follow-up, as summarized in the Central Illustration.

### Device Pocket

#### Pocket Biology

 The device pocket is a high-risk environment for infection. Following implantation, the body’s inflammatory response to the foreign material initiates a healing process that leads to the formation of a fibrous capsule around the device. This tissue has limited blood supply, reducing the delivery of immune cells and antibiotics to the pocket. As a result, bacteria that attach to the device surface can form biofilms that are difficult for both the immune system and antimicrobial therapy to eliminate, allowing infection to persist and progress [16].

#### Pocket Creation

The device should be positioned in the infraclavicular fossa (Mohrenheim), ideally on the muscular fascia, to avoid interference with shoulder mobility. The skin incision should follow natural tension lines in a medio-cranial to latero-caudal direction over this region. Pocket dissection should be atraumatic, employing a PEAK plasma blade [72] or minimalist electrosurgery with scissors to minimize thermal injury to adjacent tissues and reduce hematoma formation.

Vascular access should preferentially employ cephalic or axillary vein isolation over subclavian venipuncture [73]. For multi lead systems, each lead should enter the heart via separate venous access rather than multiple leads sharing a single sheath, optimizing lead longevity and facilitating future revision without disturbing established lead positions [74].

Leads must be secured to the muscular fascia with non-absorbable suture to prevent migration or coiling. The generator should be positioned directly over the secured leads, ensuring neither tension nor compression, and similarly anchored to the fascia [4].

#### Dead-Space Minimization

Meticulous layered closure minimizes dead space, reducing the volume available for hematoma formation and subsequent infection [75]. After lead and generator placement, the fascial layer should be approximated with a running absorbable suture in a continuous fashion. The subcutaneous fat layer is then approximated with absorbable suture in an interrupted fashion, and the skin is closed in a running intradermal fashion with non-absorbable suture for strength and optimal cosmesis.

### Pocket Level Antimicrobial Concepts

#### Pocket and Hardware Immersion

The concept of pre-implantation hardware saturation addresses a discrete infection risk window: the period between device removal from sterile packaging and final placement, during which exposed device components encounter environmental and operator-patient-mediated contamination. Preoperative immersion of the generator, leads, and connector block in an antimicrobial solution theoretically creates a residual protective coating on device surfaces that persists through initial surgical manipulation and positioning [76].

The mechanistic rationale rests on three observations: first, bacterial colonization of implanted hardware begins within the first 60 min of implantation and is most susceptible to disruption before biofilm maturation [11]; second, the device pocket’s avascular fibrous capsule (in case of generator substitution) limits systemic antibiotic penetration, making pre-implantation saturation a pharmacokinetically favorable approach to achieve high local antimicrobial concentrations at the implant-tissue interface [16]; third, hardware contamination during the operative field exposure phase, whether from airborne particles, gloved-hand contact, or instrument manipulation, establishes the initial pathogen inoculum that subsequently seeds biofilm formation [26].

Preoperative immersion differs mechanistically and temporally from post-placement pocket treatment. Post-implant irrigation occurs after device placement and addresses the pocket tissue cavity itself, attempting to reduce bacterial burden in the surrounding soft tissues or the fibrous pocket. Pre-implant saturation targets the device surface during its pre-placement, highest-exposure interval. These are complementary rather than redundant strategies, and their efficacy should be assessed independently.

Taurolidine containing adjunct device preparation has been evaluated in single-center and multicenter observational studies and appears safe, biocompatible, and antimicrobially active [77, 78]. However, its clinical effectiveness has not yet been proven because randomized controlled trials are lacking. Laboratory and in vivo studies show that taurolidine remains active on device surfaces after treatment, suggesting that its antimicrobial effect may extend beyond the implantation procedure [76, 79, 80].

Chlorhexidine-based pocket irrigation has been used, often as part of institutional protocol rather than formal research [81]. The evidence base consists of small comparative series and single-center audits, generally showing low infection rates but lacking a contemporary RCT. Chlorhexidine’s broad-spectrum activity make it a reasonable antiseptic adjunct, however, CHX based solutions require removal after immersion and are not certified for employment on CIED hardware.

Povidone-iodine pocket irrigation has been employed in some centers, though concerns about iodine absorption, potential thyroid effects with repeated procedures, and rare hypersensitivity reactions have limited widespread adoption. Available evidence suggests no effect on CIED infection rates and PVP-iodine solutions are noncertified for employment on CIED hardware.

Antibiotic pocket irrigation has been used in some centers as an adjunct to systemic pre-procedural prophylaxis. Although observational studies and a meta-analysis have suggested a reduction in pocket infection, these data are heterogeneous and predominantly non-randomized [82]. The PADIT trial provided the most robust assessment of an intensified antimicrobial strategy incorporating intraprocedural bacitracin pocket irrigation, together with additional systemic antibiotics post-procedurally (i.e., therapy). It did not demonstrate a statistically significant reduction in CIED infection compared with standard intravenous pre-procedural cefazolin [63]. Subsequent observational data have likewise failed to demonstrate a clear prophylactic advantage of antibiotic irrigation over saline [83]. Consequently, routine antibiotic pocket irrigation cannot currently be recommended as an evidence-based standard preventive measure.

The biological rationale for antibiotic pocket irrigation is limited by the transient exposure achieved by a single intraoperative admission. In contrast to an antimicrobial envelope or other sustained-release technology, pocket irrigation does not maintain antimicrobial concentrations above the minimum inhibitory concentration during the subsequent period in which bacterial adhesion, proliferation and biofilm development may occur.

*Gentamicin-impregnated collagen sponges* represent a different local antimicrobial approach, providing a biodegradable matrix intended for local hemostasis with incidental sustained local antibiotic exposure. Notably, their regulatory approval (CE marking) covers hemostatic use only; their application for infection prophylaxis in CIED procedures is therefore off-label and has not been formally certified or evaluated by regulatory bodies for this specific indication. Although efficacy for infection reduction has been investigated in other surgical settings, evidence specific to CIED implantation remains limited to observational data. Retrospective CIED cohorts have reported associations with lower infection rates and pocket hematoma, and a recent exploratory cohort reported no infections during 12-month follow-up [84–86]. Given the absence of a certified infection-prevention indication and the exclusively retrospective observational evidence base, gentamicin-collagen sponges should currently be regarded as an off-label, emerging adjunct rather than an established or regulator-endorsed infection-prevention strategy in CIED procedures.

#### Antibacterial Envelope

The WRAP-IT trial demonstrated a significant reduction in major CIED infection in patients receiving an absorbable antibiotic-eluting envelope during first implant or generator replacement. The absolute risk reduction was 0.5% (NNT ≈ 200 across the full cohort over 12 months), with greater benefit evident in higher-risk subgroups (diabetes, CKD, anticoagulation). This remains the only large-scale RCT of a device-directed anti-infection intervention and forms the foundation of current guideline recommendations for envelope use, particularly in high-risk patients.

### Post-procedural wound management

#### Immediate Post-Procedural Phase (First 3 h)

Following closure, the incision should be cleaned and disinfected with chlorhexidine in 70% alcohol and allowed to dry completely. A sterile dressing is then applied, and a pressure dressing or sandbag is placed directly over the wound site for 3 h (extended to 6 h in patients receiving oral anticoagulation) to minimize hematoma formation.

#### Patient Monitoring and Activity Restrictions (Days 1–10)

Patients should be instructed to observe the wound daily for signs of infection (purulent drainage, increasing warmth, erythema, or swelling) and to keep the wound dry during this initial healing phase. Showering is permitted only with a waterproof sterile dressing in place; full submersion bathing, swimming, and strenuous shoulder movement should be avoided. The wound dressing should be changed with antiseptic (70% chlorhexidine-alcohol) applied during each change. Non-absorbable sutures (skin-level) are typically removed at 10–14 days.

#### Patient Counsel (Weeks 2–6)

After suture removal, patients should continue to observe the generator pocket for pain, swelling, drainage, or systemic signs of infection (fever, malaise) and report promptly to their physician. Strenuous physical activity, contact sports, and full submersion bathing should be avoided for 6 weeks post-operatively to allow complete wound maturation.

All patients must inform any healthcare provider of their implanted device and should carry identification documenting the device type and manufacturer!

## Device Simplification

Increasing attention has been directed toward device simplification as a strategy to mitigate the risk of CIED infection. The underlying principle is straightforward: the most effective device-related infection may be the one that cannot occur because susceptible intravascular hardware was never implanted. By minimizing transvenous leads, reducing intravascular material burden, or avoiding the vascular space altogether, device technologies decrease both the incidence and consequences of device-related infections.

Leadless pacing systems represent the most established example of this concept. Contemporary systems now extend beyond single-chamber ventricular pacing to dual-chamber leadless platforms, broadening the population that may benefit from a completely intracardiac pacing solution. Although infections of leadless devices can occur, reported rates remain exceedingly low, likely reflecting the absence of a surgical pocket and the markedly reduced surface area available for colonization.

Similarly, conduction system pacing may indirectly contribute to infection prevention in selected patients by enabling physiologic pacing with a single lead and potentially reducing the need for more complex multi-lead systems, that require more time for manipulation during placement. While infection risk is primarily determined by overall hardware burden rather than pacing site itself, strategies that simplify device architecture may confer incremental benefits.

Defibrillation technologies have undergone a parallel evolution. The S-ICD eliminates transvenous leads entirely, thereby avoiding bloodstream exposure and the risk of lead-associated infective endocarditis [87]. Although pocket infections may still occur, infectious complications are generally confined to the extrathoracic system and can often be managed without the challenges associated with transvenous lead extraction. More recently, the extravascular (EV) ICD has emerged as a promising alternative that provides antitachycardia pacing capabilities while maintaining an entirely extravascular lead position, combining therapeutic versatility with reduced intravascular infection risk [35].

The concept of hardware minimization is further advanced by modular pacing-defibrillation systems, which integrate leadless pacing devices with extravascular or subcutaneous defibrillation platforms. These systems seek to provide the benefits of both pacing and defibrillation while avoiding transvenous leads. Looking ahead, fully leadless CRT platforms may further expand this paradigm by delivering multisite pacing without conventional transvenous lead implantation [88].

Although long-term comparative data remain limited and device selection must continue to be guided by clinical indications, these technologies collectively illustrate a broader shift toward infection-conscious device design.

## Emerging Technologies and Future Directions

Despite significant advances in procedural techniques, antimicrobial prophylaxis, and device design, CIED infection remains a persistent clinical challenge. Future preventive strategies are increasingly focused on individualized risk assessment, continuous surveillance, and biomaterial innovations that may further reduce susceptibility to microbial colonization. The convergence of artificial intelligence, digital health technologies, and novel anti-infective materials has the potential to transform infection prevention from a predominantly procedural intervention into a continuous, patient-specific process spanning the entire device lifecycle.

Artificial intelligence and machine-learning approaches are emerging as promising tools for improving infection risk stratification. Current risk scores provide valuable guidance but are limited by their reliance on predefined variables and static risk estimates. In contrast, machine-learning models can integrate large volumes of clinical, procedural, laboratory, and device-related data to identify complex interactions that may not be captured by conventional statistical methods. Such approaches may facilitate more accurate prediction of infection risk. Integration of electronic health record data with real-time procedural variables may further enhance predictive performance and support clinical decision-making at the point of care.

Digital health technologies are also expanding opportunities for earlier detection of infectious complications. Remote monitoring has become an integral component of contemporary CIED management and may evolve beyond arrhythmia and device surveillance to include infection-focused applications (i.e., frequent photos of the device pocket) [89]. Continuous assessment of device function, physiologic parameters, and patient-reported symptoms could help identify early signals of infection before overt clinical manifestations develop [90]. Coupled with smartphone-based applications and telemedicine platforms, remote monitoring may facilitate timely evaluation of wound healing and prompt intervention when concerning findings arise.

Digital wound surveillance represents an area of development. Advances in image analysis and AI-assisted pattern recognition may enable automated assessment of incision sites through patient-acquired photographs. Algorithms trained to detect erythema, swelling, wound dehiscence, or evolving pocket abnormalities could support early identification of complications while reducing the need for routine in-person follow-up visits. Such approaches may prove especially valuable in high-risk patients and in healthcare systems with limited access to specialized device clinics.

Material science innovations offer another avenue for infection prevention [15]. Next-generation antibacterial envelopes are being developed with enhanced antimicrobial properties, improved biocompatibility, and more controlled drug-release kinetics. Beyond currently available technologies, “smart” envelopes capable of responding to local inflammatory or infectious signals may provide targeted antimicrobial delivery during periods of highest risk.

Biological extracellular matrix technologies also continue to evolve. Beyond their potential role in reducing fibrosis and facilitating future device revisions, these materials may serve as platforms for localized delivery of antimicrobial agents or bioactive compounds that enhance host immune responses. Although most of these technologies remain investigational, they represent an important shift from reactive infection management toward proactive prevention of bacterial colonization. Future studies will need to determine whether these innovations can provide durable antimicrobial protection without compromising device performance, tissue integration, or long-term safety.

Novel antimicrobial polymers and surface modifications are predominantly researched for prosthetic joint replacement therapy [91]. These technologies aim to prevent bacterial adhesion and biofilm formation, the critical early steps in CIED infection pathogenesis. Strategies under investigation include anti-adhesive coatings, contact-killing surfaces, biofilm-disrupting materials, and polymer matrices capable of sustained antimicrobial release. By targeting microbial colonization before biofilm maturation occurs, these approaches may complement systemic antibiotic prophylaxis and reduce the vulnerability of implanted hardware to infection.

Nanostructured coatings and nanoparticle-based delivery systems may enable localized and prolonged antimicrobial activity at the device - tissue interface. Antimicrobial peptides, and other nanoscale platforms have demonstrated promising anti-biofilm properties in preclinical studies. Additionally, nanotechnology-based CIED adjacent sensors may eventually permit real-time detection of microbial colonization or local inflammatory responses, creating opportunities for earlier intervention before clinical infection becomes established.

Collectively, these developments could support a transition toward precision prevention as an individualized approach that integrates patient-specific risk profiling, advanced surveillance technologies, and targeted preventive interventions. Rather than applying uniform strategies to all patients, future infection prevention paradigms may dynamically adapt according to evolving risk throughout the pre-implant, peri-procedural, and post-implant phases (Table 3). Although many of these technologies remain investigational, they highlight a future in which predictive analytics, connected health systems, and bioengineered device platforms work synergistically to reduce the burden of CIED infection and improve long-term patient outcomes.

**Table 3:** Established, emerging, and investigational strategies for prevention of cardiac implantable electronic device infection

Phase	Intervention	Mechanism	Evidence	Current role
Pre-procedural	Appropriate patient selection	Avoid unnecessary implants	High	Standard
	Delay implantation during active infection	Eliminate pre-existing bacterial burden	High	Standard
	Intravenous antibiotic prophylaxis	Reduce perioperative inoculum	High	Standard
	Staphylococcal screening/decolonization	Reduce endogenous colonization	Moderate	Selected patients
	Antithrombotic optimization	Prevent hematoma	High	Standard
Intra-procedural	Alcoholic chlorhexidine	Skin antisepsis	High	Standard
	Maximum sterile barrier precautions	Prevent contamination	High	Standard
	Meticulous hemostasis	Prevent hematoma	High	Standard
	Minimize procedure duration	Reduce tissue trauma/exposure	Moderate	Standard principle
	Avoid temporary pacing	Remove major infection risk	Moderate–High	Recommended
	Ultrasound-guided venous access	Reduce vascular complications	Moderate	Increasingly adopted
	Antibacterial envelope	Prevent bacterial adhesion/biofilm	High	High-risk patients
	Iodophor incision drapes	Reduce wound contamination	Moderate-High	clinical infection endpoint underpowered
	Taurolidine immersion	Local antimicrobial effect	Low–Moderate	Investigational
	TXA-assisted hemostasis	Prevent hematoma	Low–Moderate	Emerging
	Directed HEPA airflow	Reduce airborne contamination	Low	Investigational
Device selection	Leadless pacemaker	Eliminate pocket/leads	Moderate	Selected patients
	S-ICD/EV-ICD	Avoid intravascular hardware	Moderate	Selected patients
Post-procedural	Avoid prolonged antibiotics	No additional benefit	High	Guideline-supported
	Remote wound surveillance	Early complication detection	Low	Emerging
	NPWT	Promote wound healing	Low	Selected complex revisions
Future directions	ECM envelopes	Tissue remodeling	Low	Investigational
	AI-guided risk prediction	Personalized prevention	Low	Future

Preventive interventions are categorized according to their point of application within the patient pathway, proposed mechanism of action, supporting evidence base, and current clinical role

*AI *Artificial intelligence, *CIED *Cardiac implantable electronic device, *ECM *Extracellular matrix, *EV*-*ICD *Extravascular implantable cardioverter-defibrillator, *NPWT *Negative-pressure wound therapy, *S-ICD* Subcutaneous implantable cardioverter-defibrillator, *TXA *Tranexamic acid

## Future Clinical Trials and Research Priorities

Despite significant advances in infection prevention, several important evidence gaps remain. Future clinical trials should prioritize high-risk patient populations, including individuals undergoing generator replacement, device upgrade procedures, lead revision, chronic immunosuppression, and renal replacement therapy. Additional randomized studies are needed to clarify the role of emerging pocket-management strategies, including antimicrobial irrigation solutions, biological envelopes, advanced hemostatic technologies, and novel wound-closure approaches. Furthermore, comparative effectiveness studies evaluating leadless, subcutaneous, and extravascular systems specifically from an infection-prevention perspective are warranted. The next generation of research should move beyond isolated interventions and focus on integrated infection-prevention pathways that combine individualized risk prediction, optimized procedural techniques, targeted antimicrobial strategies, and digital surveillance technologies. Such comprehensive approaches may provide the greatest opportunity to reduce the global burden of CIED infection while improving both clinical outcomes and healthcare resource utilization.

Tranexamic acid, incision drapes and skin antisepsis have been researched for CIED placement procedures and thus informing practice [27, 29, 30, 92]. Many other aspects deserve further research. Current research gaps are outlined in Table 4:

**Table 4:** Summarizes potentially modifiable procedural, operator-related, and workflow-associated interventions that may influence the risk of CIED infection but remain inadequately investigated

Intervention	Current evidence	RCT	Evidence level	Research priority
No-touch device implantation technique	Described by experienced operators; no standardized protocol	✖	Expert opinion	★★★★★
Mandatory glove change immediately before handling the generator/leads	Widely advocated; extrapolated from orthopedics and vascular surgery	✖	Surgical extrapolation	★★★★★
Double-gloving vs. single-gloving	Recommended by some infection-control experts	✖	Non-CIED surgical data	★★★★☆
Generator exposure time	Never quantified as infection determinant	✖	None	★★★★☆
Bearded vs. non bearded interventionalist	Researched predominantly for orthopedic prosthetic joint replacement	✖	Surgical extrapolation	★★★★☆
Pocket creation (atraumatic, scissors, electrosurgery devices, plasma blade)	Non standardized	✖	None	★★★★☆
Pocket retractors or wound protectors	Common in other surgery	✖	None	★★★★☆
Systematic saline irrigation protocol (volume, pressure, timing)	Saline commonly used but not researched	✖	Practice variation	★★★★☆
Antiseptic pocket irrigations (non-licensed)	case series at best	✖	Very low	★★★★☆
Topical hemostatic agents for infection prevention	Hematoma prevention studied; infection endpoint absent	✖	Indirect	★★★★☆
Energy-based hemostasis protocols	No CIED comparison	✖	None	★★★★☆
Standardized layered closure aimed at dead-space elimination	Closure studies exist but focus on cosmesis/dehiscence rather than infection	✖	Low	★★★★☆
Operator infection-prevention checklist	No multicenter implementation study	✖	Institutional experience	★★★★★
Intraoperative workflow bundle (sequence of implantation steps)	Never formally studied	✖	None	★★★★★
Restriction of door openings / laboratory traffic	Strong surgical rationale	✖	None	★★★★☆
Airflow optimization	Never evaluated	✖	None	★★★☆☆
Dedicated CIED trays	No published evaluation	✖	None	★★★☆☆
Dedicated CIED implantation teams	Suggested by quality-improvement literature	✖	None	★★★★☆
Ultrasound-guided venous access as infection intervention	Studied for pneumothorax/vascular complications, not infection	✖	Indirect	★★★☆☆
Negative-pressure dressings after high risk CIED procedures	Pilot experience only	✖	None	★★★★☆
AI-guided individualized procedural prevention bundles	Concept only	✖	None	★★★☆☆

For each intervention, the current evidence base, availability of RCT data, overall level of evidence, and proposed research priority are presented. Research priorities reflect the potential clinical impact, biological plausibility, feasibility of investigation, and the magnitude of the existing evidence gap. Notably, many interventions are currently supported only by expert opinion, institutional practice, or extrapolation from other surgical specialties, highlighting substantial opportunities for future prospective and randomized studies

*AI* Artificial intelligence, *CIED *Cardiac implantable electronic device, *RCT *Randomized controlled trial

## Conclusions

Despite substantial advances in device technology and procedural practice, CIED infection remains one of the most consequential complications of contemporary electrophysiology. Current preventive strategies have significantly reduced infection rates, yet residual risk persists, particularly among patients undergoing complex procedures, device revisions, or repeat interventions.

The future of CIED infection prevention is likely to be increasingly individualized rather than uniformly applied. Emerging risk-stratification tools, including PADIT and BLISTER [63, 68], provide a framework for tailoring preventive measures according to patient- and procedure-specific risk profiles, intended to enable more selective deployment of intensified prophylactic strategies, enhanced surveillance, and adjunctive technologies.

Progress in the field will require integration of insights from complementary research platforms. In vitro biofilm models, translational preclinical studies, device-surface investigations, and prospective clinical registries each address distinct aspects of infection pathogenesis, ranging from bacterial adhesion and biofilm development to host responses and clinical outcomes. Bridging these mechanistic and clinical perspectives remains essential for the development of more effective preventive interventions.

Increasing recognition of CIED infection as a specialized form of implant-associated infection also carries important procedural implications. Similar to other implant-based disciplines, meticulous attention to operative technique, pocket management, contamination control, and hematoma prevention should be regarded as fundamental components of infection prevention. In particular, prevention of pocket hematoma remains among the most consistently modifiable risk factors identified to date.

At a mechanistic level, the device - tissue interface represents a critical target for future investigation. The sequence of protein adsorption, bacterial attachment, and biofilm maturation ultimately determines whether contamination progresses to established infection. Improved understanding of these processes may facilitate a transition from predominantly empirical preventive strategies toward approaches specifically designed to interrupt bacterial colonization and biofilm formation.

Importantly, infection prevention extends beyond the implantation procedure itself. Patient education, structured wound surveillance, and early recognition of postoperative complications remain integral components of comprehensive infection-prevention pathways.

Collectively, emerging technologies and preventive concepts suggest a gradual shift from merely reducing infection risk toward minimizing the biological and procedural conditions that permit device colonization and biofilm establishment. Continued translational research and prospective clinical evaluation will be essential to determine which of these strategies can meaningfully improve outcomes in routine clinical practice.

## Data Availability

No datasets were generated or analysed during the current study.

## Key References

- Baldauf, B., et al., Trends in cardiac implantable electronic device infections: 2015 to 2019. BMC Cardiovascular Disorders, 2026. 26(1): p. 503.
  - ○ Findings from this study demonstrate the contemporary epidemiological burden and temporal trends of CIED infection and provide important real-world context for the continuing need for effective prevention strategies.
- Maclean, E., et al., The BLISTER Score: A Novel, Externally Validated Tool for Predicting Cardiac Implantable Electronic Device Infections, and Its Cost-Utility Implications for Antimicrobial Envelope Use. Circ Arrhythm Electrophysiol, 2024. 17(3): p. e012446.
  - ○ This study provides an externally validated risk-stratification tool that may help identify patients at increased risk of CIED infection and support more targeted, cost-conscious deployment of preventive interventions.
- Da Costa, A., et al., Chlorhexidine vs Povidone-Iodine Alcohol Solutions for Cardiac Implantable Electronic Devices. JACC, 2026. 87(2): p. 200-211.
  - ○ The CHLOVIS trial provides contemporary randomized evidence that alcoholic chlorhexidine skin disinfection did not significantly reduce CIED infection compared with alcoholic povidone-iodine skin disinfection (relative risk reduction 25%; absolute risk reduction 1%), highlighting the uncertainty surrounding established peri-procedural skin-disinfection practice

## Declarations

### Use of Artificial Intelligence

Artificial intelligence (AI)-based tools, including software incorporating AI-assisted features, were used for language and grammar editing and to assist with the refinement and finalization of Fig. 1 and the central illustration, which were designed by the authors. AI was not used to generate scientific content, analyze or interpret data, create tables, or formulate scientific conclusions. All AI-assisted outputs were reviewed, edited, and approved by the authors, who take full responsibility for the accuracy, integrity, and final content of the manuscript and its figures.
